# 2,2,4-Trimethyl-7-nitro-2,3-dihydro-1*H*-1,5-benzodiazepin-5-ium perchlorate

**DOI:** 10.1107/S1600536810024475

**Published:** 2010-06-26

**Authors:** Sayed Hasan Mehdi, Othman Sulaiman, Raza Murad Ghalib, Chin Sing Yeap, Hoong-Kun Fun

**Affiliations:** a School of Industrial Technology, Universiti Sains Malaysia, 11800 USM, Penang, Malaysia; b X-ray Crystallography Unit, School of Physics, Universiti Sains Malaysia, 11800 USM, Penang, Malaysia

## Abstract

In the title mol­ecular salt, C_12_H_16_N_3_O_2_^+^·ClO_4_^−^, the nitro group is close to being coplanar with the benzene ring [dihedral angle = 8.1 (3)°]. The seven-membered ring has a maximum deviation of 0.502 (3) Å at the C atom between the dimethyl- and methyl-substituted C atoms. In the crystal, the components are linked into infinite sheets lying parallel to the *bc* plane by N—H⋯O and C—H⋯O hydrogen bonds. A short O⋯N contact of 2.896 (4) Å occurs within the sheets and a short O⋯O contact of 2.608 (4) Å occurs between the sheets.

## Related literature

For general background and applications of benzimidazole derivatives, see: Landquist (1984 ▶); Insuasty *et al.* (2010 ▶); Balakrishna & Kaboudin (2001 ▶); Ballo *et al.* (2010 ▶). For the preparation of the title compound, see: Grech *et al.* (1994 ▶). For ring conformations, see Cremer & Pople (1975 ▶). For the stability of the temperature controller used for the data collection, see: Cosier & Glazer (1986 ▶).
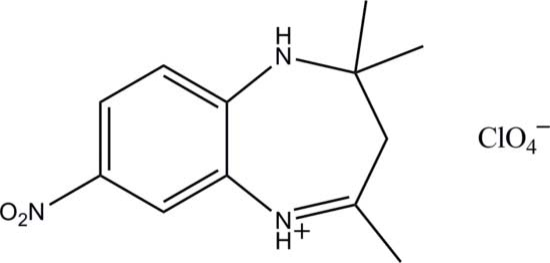


## Experimental

### 

#### Crystal data

C_12_H_16_N_3_O_2_^+^·ClO_4_^−^
*M* *_r_* = 333.73
Monoclinic, 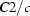

*a* = 21.046 (7) Å
*b* = 11.818 (3) Å
*c* = 15.636 (6) Å
β = 132.176 (9)°
*V* = 2882.0 (16) Å^3^
*Z* = 8
Mo *K*α radiation
μ = 0.30 mm^−1^
*T* = 100 K
0.30 × 0.17 × 0.08 mm

#### Data collection

Bruker APEXII DUO CCD diffractometer
Absorption correction: multi-scan (*SADABS*; Bruker, 2009 ▶) *T*_min_ = 0.915, *T*_max_ = 0.976
24582 measured reflections
3323 independent reflections
2816 reflections with *I* > 2σ(*I*)
*R*_int_ = 0.066

#### Refinement

*R*[*F* ^2^ > 2σ(*F* ^2^)] = 0.046
*wR*(*F* ^2^) = 0.138
*S* = 1.05
3323 reflections
210 parameters
H atoms treated by a mixture of independent and constrained refinement
Δρ_max_ = 0.69 e Å^−3^
Δρ_min_ = −0.69 e Å^−3^

### 

Data collection: *APEX2* (Bruker, 2009 ▶); cell refinement: *SAINT* (Bruker, 2009 ▶); data reduction: *SAINT*; program(s) used to solve structure: *SHELXTL* (Sheldrick, 2008 ▶); program(s) used to refine structure: *SHELXTL*; molecular graphics: *SHELXTL*; software used to prepare material for publication: *SHELXTL* and *PLATON* (Spek, 2009 ▶).

## Supplementary Material

Crystal structure: contains datablocks global, I. DOI: 10.1107/S1600536810024475/hb5507sup1.cif

Structure factors: contains datablocks I. DOI: 10.1107/S1600536810024475/hb5507Isup2.hkl

Additional supplementary materials:  crystallographic information; 3D view; checkCIF report

## Figures and Tables

**Table 1:** Hydrogen-bond geometry (Å, °)

*D*—H⋯*A*	*D*—H	H⋯*A*	*D*⋯*A*	*D*—H⋯*A*
N1—H1*N*1⋯O6^i^	0.80 (3)	2.15 (3)	2.941 (3)	173 (3)
N2—H1*N*2⋯O4	0.82 (5)	2.09 (4)	2.864 (4)	156 (4)
N2—H1*N*2⋯O4^ii^	0.82 (5)	2.46 (5)	3.000 (4)	124 (3)
C3—H3*A*⋯O1^i^	0.93	2.51	3.373 (3)	155
C11—H11*A*⋯O5^i^	0.96	2.58	3.524 (3)	169
C11—H11*B*⋯O3^iii^	0.96	2.45	3.396 (3)	168

Symmetry codes: (i) 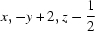; (ii) 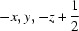; (iii) 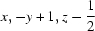.

**Fig. 1.**
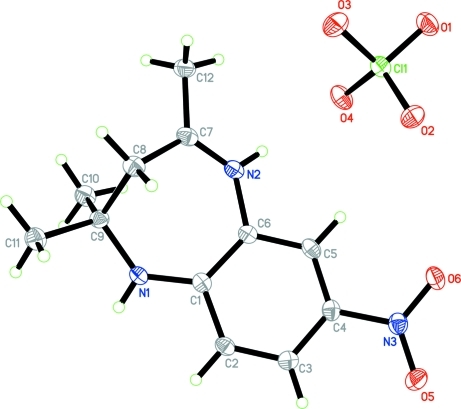
The molecular structure of (I) with 50% probability ellipsoids for non-H atoms.

**Fig. 2.**
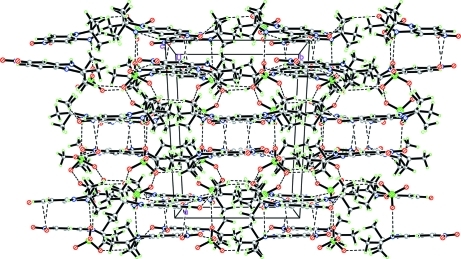
The crystal packing of (I), viewed down the c axis, showing the components linked into a 3-D network. Intermolecular hydrogen bonds are shown as dashed lines.

## supplementary crystallographic information

### Comment

Benzodiazepines are interesting compounds due to their wide range of biological activities (Landquist, 1984). Recently many methods have been employed for the synthesis of benzodiazepines derivatives (Insuasty *et al.*, 2010; Balakrishna & Kaboudin, 2001; Ballo *et al.*, 2010). Here we report the synthesis and the crystal structure of title compound.

The asymmetric unit of title compound (Fig. 1) consists of one the benzodiazepinium cation and one perchlorate anion. The nitro group is coplanar with the benzene ring with the dihedral angle of 8.1 (3)°. The seven-membered ring (N1/C1/C6/N2/C7–C9) have a maximum deviation of 0.502 (3) Å at atom C8. In the crystal structure, the molecules are linked into infinite two-dimensional planes parallel to *bc* plane by the intermolecular N—H···O, C—H···O hydrogen bonds (Table 1) and short O6···N3 interaction of 2.896 (4) Å. Short O2···O3 interaction of 2.608 (4) Å linked these planes into a three-dimensional framewrok (Fig. 2).

### Experimental

A mixture of 4-nitro *o*-phenylenediamine (0.153 g m) and 4-hydroxy coumarin (0.162 g m) in molar ratio 1:1 was refluxed in a mixture of acetic acid-ethanol (1:1 *v*/*v*) for 3 h (Grech *et al.*, 1994). The solid settled in the reaction mixture was filtered and crystallized from ethanol to furnish brownish plates of the unexpected title compound, (I) (55%, m.p. 458 K).

### Refinement

The N-bound hydrogen atoms were located from the difference Fourier map and were refined freely. The rest of hydrogen atoms were positioned geometrically [C–H = 0.93–0.97 Å] and refined using a riding model, with *U*_iso_(H) = 1.2 or 1.5*U*_eq_(C). Rotating-group model was applied for methyl groups.

### Crystal data

**Table:**

C_12_H_16_N_3_O_2_^+^·ClO_4_^−^	*F*(000) = 1392
*M**_r_* = 333.73	*D*_x_ = 1.538 Mg m^−^^3^
Monoclinic, *C*2/*c*	Mo *K*α radiation, λ = 0.71073 Å
Hall symbol: -C 2yc	Cell parameters from 7331 reflections
*a* = 21.046 (7) Å	θ = 2.2–29.8°
*b* = 11.818 (3) Å	µ = 0.30 mm^−^^1^
*c* = 15.636 (6) Å	*T* = 100 K
β = 132.176 (9)°	Plate, brown
*V* = 2882.0 (16) Å^3^	0.30 × 0.17 × 0.08 mm
*Z* = 8	

### Data collection

**Table:**

Bruker APEXII DUO CCD diffractometer	3323 independent reflections
Radiation source: fine-focus sealed tube	2816 reflections with *I* > 2σ(*I*)
graphite	*R*_int_ = 0.066
φ and ω scans	θ_max_ = 27.5°, θ_min_ = 2.2°
Absorption correction: multi-scan (*SADABS*; Bruker, 2009)	*h* = −27→27
*T*_min_ = 0.915, *T*_max_ = 0.976	*k* = −15→15
24582 measured reflections	*l* = −20→20

### Refinement

**Table:**

Refinement on *F*^2^	Primary atom site location: structure-invariant direct methods
Least-squares matrix: full	Secondary atom site location: difference Fourier map
*R*[*F*^2^ > 2σ(*F*^2^)] = 0.046	Hydrogen site location: inferred from neighbouring sites
*wR*(*F*^2^) = 0.138	H atoms treated by a mixture of independent and constrained refinement
*S* = 1.05	*w* = 1/[σ^2^(*F*_o_^2^) + (0.0714*P*)^2^ + 7.0069*P*] where *P* = (*F*_o_^2^ + 2*F*_c_^2^)/3
3323 reflections	(Δ/σ)_max_ < 0.001
210 parameters	Δρ_max_ = 0.69 e Å^−^^3^
0 restraints	Δρ_min_ = −0.68 e Å^−^^3^

### Special details

**Table:**

Experimental. The crystal was placed in the cold stream of an Oxford Cryosystems Cobra open-flow nitrogen cryostat (Cosier & Glazer, 1986) operating at 100.0 (1) K.
Geometry. All e.s.d.'s (except the e.s.d. in the dihedral angle between two l.s. planes) are estimated using the full covariance matrix. The cell e.s.d.'s are taken into account individually in the estimation of e.s.d.'s in distances, angles and torsion angles; correlations between e.s.d.'s in cell parameters are only used when they are defined by crystal symmetry. An approximate (isotropic) treatment of cell e.s.d.'s is used for estimating e.s.d.'s involving l.s. planes.
Refinement. Refinement of *F*^2^ against ALL reflections. The weighted *R*-factor *wR* and goodness of fit *S* are based on *F*^2^, conventional *R*-factors *R* are based on *F*, with *F* set to zero for negative *F*^2^. The threshold expression of *F*^2^ > σ(*F*^2^) is used only for calculating *R*-factors(gt) *etc*. and is not relevant to the choice of reflections for refinement. *R*-factors based on *F*^2^ are statistically about twice as large as those based on *F*, and *R*- factors based on ALL data will be even larger.

### Fractional atomic coordinates and isotropic or equivalent isotropic displacement parameters (Å^2^)

**Table:**

	*x*	*y*	*z*	*U*_iso_*/*U*_eq_	
Cl1	0.15021 (3)	0.71202 (4)	0.49658 (4)	0.01923 (16)	
O1	0.13150 (11)	0.68718 (14)	0.56727 (14)	0.0259 (4)	
O2	0.19403 (11)	0.82394 (13)	0.52807 (14)	0.0245 (4)	
O3	0.21587 (10)	0.62367 (14)	0.52533 (14)	0.0256 (4)	
O4	0.07604 (10)	0.70691 (14)	0.37336 (13)	0.0234 (4)	
O5	0.08273 (11)	1.21240 (13)	0.25797 (14)	0.0249 (4)	
O6	0.09384 (11)	1.07773 (14)	0.36195 (13)	0.0246 (4)	
N1	0.11724 (12)	0.79665 (15)	0.04379 (16)	0.0183 (4)	
N2	0.10204 (12)	0.72103 (15)	0.21588 (15)	0.0176 (4)	
N3	0.08881 (11)	1.11171 (16)	0.28211 (15)	0.0194 (4)	
C1	0.10956 (12)	0.86625 (17)	0.10449 (16)	0.0152 (4)	
C2	0.10763 (13)	0.98440 (18)	0.08478 (17)	0.0166 (4)	
H2A	0.1127	1.0079	0.0328	0.020*	
C3	0.09866 (12)	1.06450 (17)	0.13882 (17)	0.0166 (4)	
H3A	0.0967	1.1410	0.1232	0.020*	
C4	0.09241 (12)	1.02892 (18)	0.21822 (17)	0.0172 (4)	
C5	0.09300 (13)	0.91616 (18)	0.24014 (17)	0.0172 (4)	
H5A	0.0872	0.8945	0.2918	0.021*	
C6	0.10220 (12)	0.83451 (17)	0.18541 (17)	0.0155 (4)	
C7	0.14051 (13)	0.63366 (18)	0.21973 (17)	0.0180 (4)	
C8	0.19130 (13)	0.64237 (19)	0.18564 (18)	0.0191 (4)	
H8A	0.2184	0.5700	0.1991	0.023*	
H8B	0.2364	0.6978	0.2347	0.023*	
C9	0.13798 (13)	0.67632 (17)	0.05830 (17)	0.0165 (4)	
C10	0.05645 (14)	0.60604 (19)	−0.02308 (18)	0.0216 (4)	
H10A	0.0269	0.6260	−0.1015	0.032*	
H10B	0.0200	0.6207	−0.0077	0.032*	
H10C	0.0710	0.5271	−0.0113	0.032*	
C11	0.19389 (15)	0.6604 (2)	0.0295 (2)	0.0234 (5)	
H11A	0.1627	0.6841	−0.0485	0.035*	
H11B	0.2091	0.5821	0.0377	0.035*	
H11C	0.2450	0.7051	0.0813	0.035*	
C12	0.13540 (15)	0.52362 (18)	0.26078 (19)	0.0222 (4)	
H12A	0.0970	0.5305	0.2738	0.033*	
H12B	0.1914	0.5028	0.3315	0.033*	
H12C	0.1145	0.4665	0.2035	0.033*	
H1N1	0.1130 (16)	0.826 (2)	−0.006 (2)	0.016 (6)*	
H1N2	0.0798 (19)	0.715 (2)	0.243 (3)	0.030 (8)*	

### Atomic displacement parameters (Å^2^)

**Table:**

	*U*^11^	*U*^22^	*U*^33^	*U*^12^	*U*^13^	*U*^23^
Cl1	0.0193 (3)	0.0214 (3)	0.0170 (3)	−0.00116 (18)	0.0122 (2)	−0.00003 (18)
O1	0.0328 (9)	0.0283 (9)	0.0249 (8)	−0.0029 (7)	0.0228 (8)	0.0002 (7)
O2	0.0298 (8)	0.0167 (8)	0.0277 (8)	−0.0057 (6)	0.0195 (7)	−0.0039 (6)
O3	0.0230 (8)	0.0219 (8)	0.0289 (9)	0.0046 (6)	0.0162 (7)	0.0013 (7)
O4	0.0169 (7)	0.0350 (9)	0.0147 (8)	−0.0018 (6)	0.0091 (7)	−0.0003 (6)
O5	0.0305 (9)	0.0176 (8)	0.0253 (8)	0.0029 (6)	0.0183 (7)	0.0006 (6)
O6	0.0319 (9)	0.0267 (8)	0.0192 (8)	0.0026 (7)	0.0188 (7)	0.0002 (6)
N1	0.0239 (9)	0.0180 (9)	0.0167 (9)	0.0012 (7)	0.0152 (8)	0.0019 (7)
N2	0.0186 (8)	0.0192 (9)	0.0156 (8)	−0.0018 (7)	0.0118 (7)	0.0008 (7)
N3	0.0185 (8)	0.0213 (9)	0.0169 (8)	0.0015 (7)	0.0113 (7)	−0.0003 (7)
C1	0.0108 (8)	0.0192 (10)	0.0122 (9)	−0.0005 (7)	0.0064 (7)	−0.0002 (7)
C2	0.0147 (9)	0.0202 (10)	0.0134 (9)	−0.0019 (8)	0.0088 (8)	0.0006 (7)
C3	0.0132 (9)	0.0174 (10)	0.0136 (9)	−0.0013 (7)	0.0067 (8)	0.0001 (7)
C4	0.0146 (9)	0.0204 (10)	0.0141 (9)	0.0014 (8)	0.0087 (8)	−0.0008 (8)
C5	0.0144 (9)	0.0234 (10)	0.0127 (9)	0.0004 (8)	0.0086 (8)	0.0012 (8)
C6	0.0128 (9)	0.0179 (10)	0.0138 (9)	−0.0011 (7)	0.0082 (8)	0.0003 (7)
C7	0.0164 (9)	0.0205 (10)	0.0113 (9)	−0.0025 (8)	0.0069 (8)	0.0003 (7)
C8	0.0159 (9)	0.0220 (10)	0.0172 (10)	0.0016 (8)	0.0102 (8)	0.0027 (8)
C9	0.0162 (9)	0.0164 (9)	0.0174 (10)	0.0013 (8)	0.0115 (8)	0.0018 (8)
C10	0.0209 (10)	0.0231 (11)	0.0182 (10)	−0.0032 (8)	0.0121 (9)	−0.0019 (8)
C11	0.0254 (11)	0.0241 (11)	0.0289 (12)	0.0026 (9)	0.0215 (10)	0.0029 (9)
C12	0.0278 (11)	0.0181 (10)	0.0207 (10)	−0.0013 (9)	0.0163 (9)	0.0017 (8)

### Geometric parameters (Å, °)

**Table:**

Cl1—O1	1.4310 (16)	C4—C5	1.374 (3)
Cl1—O4	1.4538 (16)	C5—C6	1.387 (3)
Cl1—O2	1.4941 (16)	C5—H5A	0.9300
Cl1—O3	1.5388 (17)	C7—C8	1.484 (3)
O5—N3	1.229 (2)	C7—C12	1.486 (3)
O6—N3	1.246 (2)	C8—C9	1.544 (3)
N1—C1	1.345 (3)	C8—H8A	0.9700
N1—C9	1.460 (3)	C8—H8B	0.9700
N1—H1N1	0.79 (3)	C9—C10	1.524 (3)
N2—C7	1.288 (3)	C9—C11	1.528 (3)
N2—C6	1.424 (3)	C10—H10A	0.9600
N2—H1N2	0.81 (3)	C10—H10B	0.9600
N3—C4	1.436 (3)	C10—H10C	0.9600
C1—C6	1.425 (3)	C11—H11A	0.9600
C1—C2	1.425 (3)	C11—H11B	0.9600
C2—C3	1.364 (3)	C11—H11C	0.9600
C2—H2A	0.9300	C12—H12A	0.9600
C3—C4	1.398 (3)	C12—H12B	0.9600
C3—H3A	0.9300	C12—H12C	0.9600
			
O1—Cl1—O4	114.06 (10)	N2—C7—C8	120.61 (19)
O1—Cl1—O2	110.86 (10)	N2—C7—C12	119.75 (19)
O4—Cl1—O2	110.46 (10)	C8—C7—C12	119.63 (19)
O1—Cl1—O3	107.10 (10)	C7—C8—C9	113.96 (17)
O4—Cl1—O3	108.27 (10)	C7—C8—H8A	108.8
O2—Cl1—O3	105.65 (10)	C9—C8—H8A	108.8
C1—N1—C9	130.62 (18)	C7—C8—H8B	108.8
C1—N1—H1N1	115.8 (19)	C9—C8—H8B	108.8
C9—N1—H1N1	113.4 (19)	H8A—C8—H8B	107.7
C7—N2—C6	128.90 (19)	N1—C9—C10	110.47 (17)
C7—N2—H1N2	118 (2)	N1—C9—C11	106.44 (17)
C6—N2—H1N2	113 (2)	C10—C9—C11	110.21 (18)
O5—N3—O6	122.75 (18)	N1—C9—C8	109.64 (17)
O5—N3—C4	119.30 (18)	C10—C9—C8	111.79 (17)
O6—N3—C4	117.94 (18)	C11—C9—C8	108.13 (17)
N1—C1—C6	127.01 (19)	C9—C10—H10A	109.5
N1—C1—C2	116.50 (18)	C9—C10—H10B	109.5
C6—C1—C2	116.48 (18)	H10A—C10—H10B	109.5
C3—C2—C1	122.82 (19)	C9—C10—H10C	109.5
C3—C2—H2A	118.6	H10A—C10—H10C	109.5
C1—C2—H2A	118.6	H10B—C10—H10C	109.5
C2—C3—C4	118.43 (19)	C9—C11—H11A	109.5
C2—C3—H3A	120.8	C9—C11—H11B	109.5
C4—C3—H3A	120.8	H11A—C11—H11B	109.5
C5—C4—C3	121.52 (19)	C9—C11—H11C	109.5
C5—C4—N3	118.88 (18)	H11A—C11—H11C	109.5
C3—C4—N3	119.55 (19)	H11B—C11—H11C	109.5
C4—C5—C6	120.17 (19)	C7—C12—H12A	109.5
C4—C5—H5A	119.9	C7—C12—H12B	109.5
C6—C5—H5A	119.9	H12A—C12—H12B	109.5
C5—C6—N2	114.60 (18)	C7—C12—H12C	109.5
C5—C6—C1	120.55 (19)	H12A—C12—H12C	109.5
N2—C6—C1	124.83 (18)	H12B—C12—H12C	109.5
			
C9—N1—C1—C6	−14.5 (3)	C7—N2—C6—C1	31.3 (3)
C9—N1—C1—C2	166.30 (19)	N1—C1—C6—C5	−178.74 (19)
N1—C1—C2—C3	178.87 (18)	C2—C1—C6—C5	0.5 (3)
C6—C1—C2—C3	−0.5 (3)	N1—C1—C6—N2	0.2 (3)
C1—C2—C3—C4	1.0 (3)	C2—C1—C6—N2	179.44 (18)
C2—C3—C4—C5	−1.6 (3)	C6—N2—C7—C8	−2.4 (3)
C2—C3—C4—N3	175.77 (18)	C6—N2—C7—C12	176.47 (19)
O5—N3—C4—C5	−175.37 (19)	N2—C7—C8—C9	−62.3 (3)
O6—N3—C4—C5	6.1 (3)	C12—C7—C8—C9	118.8 (2)
O5—N3—C4—C3	7.1 (3)	C1—N1—C9—C10	97.6 (2)
O6—N3—C4—C3	−171.40 (18)	C1—N1—C9—C11	−142.8 (2)
C3—C4—C5—C6	1.7 (3)	C1—N1—C9—C8	−26.1 (3)
N3—C4—C5—C6	−175.70 (18)	C7—C8—C9—N1	74.8 (2)
C4—C5—C6—N2	179.81 (18)	C7—C8—C9—C10	−48.0 (2)
C4—C5—C6—C1	−1.2 (3)	C7—C8—C9—C11	−169.51 (18)
C7—N2—C6—C5	−149.7 (2)		

### Hydrogen-bond geometry (Å, °)

**Table:**

*D*—H···*A*	*D*—H	H···*A*	*D*···*A*	*D*—H···*A*
N1—H1N1···O6^i^	0.80 (3)	2.15 (3)	2.941 (3)	173 (3)
N2—H1N2···O4	0.82 (5)	2.09 (4)	2.864 (4)	156 (4)
N2—H1N2···O4^ii^	0.82 (5)	2.46 (5)	3.000 (4)	124 (3)
C3—H3A···O1^i^	0.93	2.51	3.373 (3)	155
C11—H11A···O5^i^	0.96	2.58	3.524 (3)	169
C11—H11B···O3^iii^	0.96	2.45	3.396 (3)	168

Symmetry codes: (i) *x*, −*y*+2, *z*−1/2; (ii) −*x*, *y*, −*z*+1/2; (iii) *x*, −*y*+1, *z*−1/2.
